# Road map for primary hepatocyte qualification in human liver organ models

**DOI:** 10.1186/s44330-026-00058-7

**Published:** 2026-02-09

**Authors:** Mahboubeh Varmazyad, Dillon C. Gavlock, Michael W. Castiglione, Richard DeBiasio, Gregory LaRocca, Celeste Reese, Lawrence A. Vernetti, Mark Schurdak, Andrew M. Stern, D. Lansing Taylor, Mark T. Miedel, Jacquelyn A. Brown

**Affiliations:** 1 Organ Pathobiology and Therapeutic Institute, University of Pittsburgh, Pittsburgh, PA 15261 USA; 2 Pittsburgh Liver Research Center, University of Pittsburgh, Pittsburgh, PA 15261 USA; 3 Department of Computational and System Biology, School of Medicine, University of Pittsburgh, Pittsburgh, PA 15260 USA; 4 Department of Pharmacology and Chemical Biology, University of Pittsburgh, Pittsburgh, PA 15260 USA

**Keywords:** Microphysiological systems (MPS), Primary human hepatocytes, Metabolic Dysfunction-Associated steatotic liver disease (MASLD), Cell selection framework, Liver disease modeling, Hepatocyte functional evaluation

## Abstract

**Background:**

Microphysiological systems (MPS) are powerful tools for modeling human organ function and evaluating therapeutic interventions. The performance and translational relevance of MPS are highly dependent on the quality and suitability of the cells used. Given the expanding array of primary and stem cell-derived sources, there is a critical need for systematic frameworks to guide cell selection, particularly for disease-specific applications such as Metabolic Dysfunction-Associated Steatotic Liver Disease (MASLD).

**Methods:**

We developed a phased, stepwise methodology to evaluate and select primary human hepatocytes for use in a liver MPS model of MASLD. The protocol incorporates assessments of cell source quality, plating efficiency, viability, baseline liver function, and responsiveness to disease-inducing conditions. Quantitative metrics and pass/fail criteria were applied at each stage to ensure consistent and reproducible evaluation across cell lots.

**Results:**

Application of the protocol enabled effective triaging of hepatocyte sources, distinguishing cell lots with superior plating performance and functional profiles. Selected hepatocytes exhibited robust expression of liver-specific markers, maintained metabolic activity, and demonstrated disease-relevant phenotypes under MASLD-inducing conditions.

**Discussion:**

This structured evaluation framework facilitates the identification of high-quality hepatocytes for MPS liver models, improving reproducibility and disease modeling accuracy. While this protocol was tailored for MASLD, the approach is adaptable to other liver diseases or applications. Limitations include potential variability in donor tissue availability and the need for standardization across laboratories.

**Clinical trial number:**

Not applicable.

**Supplementary Information:**

The online version contains supplementary material available at 10.1186/s44330-026-00058-7.

## Introduction

Liver (MPS), also known as liver-on-a-chip models, are advanced in vitro platforms that replicate key aspects of liver structure and function. These systems can integrate microfluidics, 3D cell cultures, and biomimetic environments to create physiologically relevant liver models for drug testing, disease modeling, ADME-TOX studies, while also serving as a mature cell model for evaluating induced pluripotent stem cell (iPSC)-derived cells [1–4]. The number of publications citing organ on a chip or microphysiological systems have been exponentially increasing over the last decade [5, 6] as the use of these platforms has been expanded to provide insights in drug safety, pharmacokinetics, acute and chronic disease modeling as well as precision medicine [7–13]. Primary liver cells will serve as a critical reference for the development of mature, maximally functional iPSC-derived cells that can serve as a stable source of heterogenous patient populations.

Metabolic dysfunction-associated steatotic liver disease (MASLD), formerly known as non-alcoholic fatty liver disease (NAFLD), is a chronic liver condition that affects approximately 30% of the global adult population [14]. Managing MASLD places a substantial financial strain on healthcare systems, impacting about 30 million adults in the United States alone, with projections suggesting this number could exceed 100 million by 2030 [15]. The high rate of failure in MASLD drug development is attributed to multiple challenges, including the absence of optimized liver models for accurate therapeutic prediction [16]. A major concern for clinicians remains the ability to distinguish likely responders from non-responders for emerging MASLD treatments [17]. Thus, a critical need is to implement highly reproducible preclinical in vitro model systems that can be utilized to monitor both MASLD progression and response to drug treatment. Addressing this gap, liver MPS offer a transformative approach to disease modeling and drug evaluation by enhancing physiological relevance and reducing reliance on animal models but their success depends critically on a robust and reproducible process for selecting and validating the cells used to populate these systems. To address these concerns, consortia of scientists and pharmaceutical researchers have developed initial benchmarks for evaluating the performance of organ-on-a-chip systems [18]. These standards offer an important starting point for system-wide comparison and reproducibility. However, they often prioritize general functional outputs, which may inadvertently exclude cell types that exhibit disease- or infection-relevant phenotypes but do not meet these broad criteria. This approach risks eliminating biologically crucial cells simply because their behavior falls outside predefined, non-contextualized metrics. Cell selection strategies should incorporate measures of disease progression, mechanistic bioactivity, and context-specific responses to ensure that evaluation is both comprehensive and biologically meaningful. Crucially, no single metric should dominate the assessment process; instead, an integrative framework is needed that balances rigor, relevance, and reproducibility. While liver MPS offer the potential to overcome many limitations of traditional models by providing a dynamic and physiologically relevant environment for hepatocytes and other liver cell types [19], their performance is ultimately dependent on the quality of the cells used. As such, refining cell sourcing and characterization in alignment with disease-specific goals is essential to fully realize the potential of liver MPS in drug discovery and disease modeling.

We have published 2 liver platforms designed to recapitulate the liver acinus organ structure and functions. The liver acinus MPS (LAMPS) is a simple one chamber device that is easy to use, single zoneed and cost-effective and the vascularized liver acinus MPS (vLAMPS) generates oxygen zonation and allows the introduction of cells such as cancer cells and immune cells in the vascular channel that can access the hepatic channel. Both models employ multiple cell types including hepatocytes, liver sinusoidal endothelial cells (LSECs), Stellate cells and Kupffer cells [4, 20, 21].

**Fig. 1:**
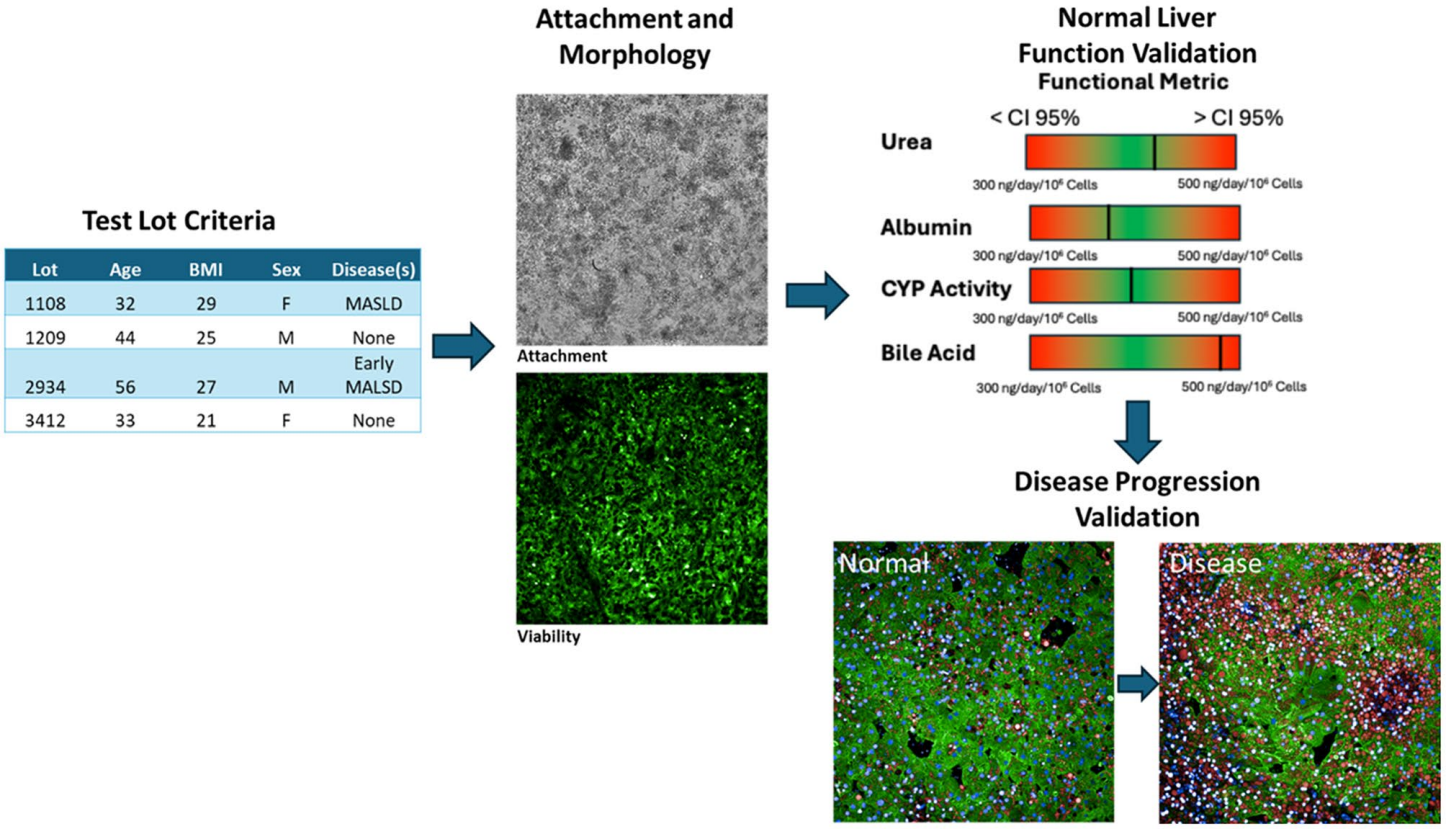
Overview of the decision pipeline for human cell lot validation and selection. **(A)** Key donor information used for test lot selection, including age, sex, BMI, and disease status. **(B)** Representative example of hepatic cell morphology and viability that meets passing criteria. **(C)** Acceptable ranges for normal liver function metrics urea, albumin, CYP activity, and bile acid production used to determine passing scores. **(D)** Representative LAMPS image used to assess and validate disease progression stage imaging of markers for steatosis and fibrosis

Multiple commercial platforms that support liver MPS have been developed. Emulate’s Human Liver-Chip replicates key in vivo liver functions by incorporating critical features such as 3D multicellular architecture and vascular flow in a two-chamber device. It provides a model that more accurately reflects human biology than traditional approaches, with a reported sensitivity of 100% and specificity of 90% for predicting drug-induced liver injury (DILI) [22]. CN Bio’s Liver-on-a-Chip: CN Bio offers predictive human liver models cultured in multi-chip liver plates using their PhysioMimix^®^ OOC microphysiological systems. These models replicate the multicellular architecture of the liver, facilitating studies on drug metabolism, transport, DILI and chronic liver diseases. The dynamic 3D microenvironment promotes cell viability and functionality, enabling long-term culture for up to four weeks additional commercially available platforms of note though not exhaustive also include Javelin and Hesperos liver MPS [23]. At present, most liver MPS platforms are cell source agnostic, requiring only a minimum number of distinct cell types to complete the model. However, despite decades of research, there is no universally optimal cell source or configuration for all experimental goals. More critically, the current state of MPS platforms lacks standardized, rigorous criteria for cell selection, which limits inter-laboratory reproducibility and hampers broader adoption of these platforms for consistent and meaningful biological insights [18]. To advance this technology beyond academic research and into clinical and pharmaceutical applications, it is essential to establish qualification standards not only for the platform itself but also for the cells used to construct the models [24, 25]. In this study, we present and validate a comprehensive process for the selection and qualification of hepatocytes used in microfluidic liver models aimed at investigating MASLD (Fig. 1). Similar approaches are now being applied to the liver non-parenchymal cells.

## Materials and methods

### Cell sources and initial culture

Human hepatocyte lots used in this paper include replatable HU8391, HU8408 HU8449, HU8442 and HU8450 (Thermo Fisher Scientific), and replatable 1183, 658, and 1045 (AnaBios). Replatable is defined as showing attachment of a confluent monolayer of cells 24 h or less after cell seeding. Liver sinusoidal endothelial cells (LSECs) were purchased from LifeNet.Health (NPC-AD-LEC-P1). The human monoblast cell line, THP-1, used to generate Kupffer-like cells, was purchased from ATCC (TIB-202). Before seeding into the LAMPS, THP-1 cells were treated 48 h in advance with 200 ng/mL phorbol 12-myristate 13-acetate (PMA; Sigma-Aldrich, 524400) to induce differentiation into macrophage-like cells and inhibit cell growth (Traore et al., 2005). LX-2 human stellate cells were obtained from Sigma-Aldrich (SCC064). LSECs were cultured in endothelial cell basal medium-2 (EBM-2; Lonza, CC-3162). THP-1 cells were maintained in suspension in RPMI 1640 medium (Cytiva, SH30096.FS) supplemented with 10% fetal bovine serum (FBS; Corning, MT35010CV), 100 µg/mL penicillin-streptomycin (Cytiva, SV30010), and 2 mM L-glutamine (Cytiva, SH30034.01). LX-2 cells were cultured in Dulbecco’s modified Eagle’s medium (DMEM; Thermo Fisher Scientific, 11965118) with 2% FBS, 100 units/mL penicillin, and 100 µg/mL streptomycin.

### LAMPS assembly and maintenance

LAMPS studies were conducted following previously established protocols for model assembly ([4, 9, 10, 26–28]; Supplementary Figure S1). A detailed description of the assembly process is provided in the Supplementary Methods. Briefly, LAMPS were built using ChipShop (Fluidic 557 Reaction Chamber Chip), incorporating four key liver cell types at the following densities: primary cryopreserved human hepatocytes (2.70–2.85 × 10⁶ cells/mL), primary LSECs (1.5 × 10⁶ cells/mL), THP-1 (0.4 × 10⁶ cells/mL), and LX-2 (0.2 × 10⁶ cells/mL). The resulting composition hepatocytes (56%), THP-1 (18%), LSECs (22%), and LX-2 (4%) aligns with previously published scaling methods [10, 26–28]. Prior to cell seeding, the device interiors were coated with 100 µg/mL bovine fibronectin (Sigma-Aldrich, F1141) and 150 µg/mL rat-tail collagen I (Corning, 354249) in PBS. To ensure full coverage of fluidic pathways, chambers, 90–100 µL of media or cell suspension was injected per device. The devices were then overlaid with 1.5 mg/mL rat-tail collagen I (Corning) and maintained under perfusion with different conditions for 8 days at a flow rate of 5 µL/h, mimicking zone-3 oxygen tension [26].

### Media formulation; normal fasting, early metabolic syndrome, and late metabolic syndrome media

The media were prepared following previously established protocols [9] to model MASLD progression from normal fasting (NF) to Early Metabolic Syndrome (EMS or early stage MASLD), and Late Metabolic Syndrome (LMS or late-stage MASLD). Cells were maintained in NF until the MPS was hooked up to flow on the day of flow initiation the MPS was then exposed to one of the 3 media types (NF, EMS, LMS) for the duration of the experiment (8 days). It has previously been established by our team that exposure to the diseased media i.e. either EMS or LMS is sufficient for establishing many of the key features of MASLD at the appropriate stage [9].

### CYP activity

A total of 50,000 primary hepatocytes were sub-cultured in William’s E Medium supplemented with 2 mM L-glutamine and 5% FBS in 96-well collagen I-coated microtiter plates and allowed to adhere overnight. The plating medium was then removed and replaced with 100 µL of either vehicle control medium (0.1% DMSO in NF, EMS, or LMS media) or Cyp3A4 induction medium (10 µM Rifampicin in 0.1% NF, EMS, or LMS media) for 48 h. Cyp3A4 activity was measured using the ProMega P450-Glo (IPA) assay kit following the manufacturer’s protocol. After 48 h induction, the medium was removed and replaced with 100 µL of NF, EMS, or LMS media containing a 1:1000 dilution of the Luciferin-IPA substrate. The plates were incubated for 4 h, after which a 50 µL aliquot of the medium was transferred to a white opaque 96-well microtiter plate. Subsequently, 50 µL of luminescent detection reagent was added, and the plate was incubated for 20 min before measuring luminescence using a SpectraMax 5 with a 0.5-second luminescence collection time.

### Bile canaliculi histology and analysis

A total of 50,000 primary hepatocytes were sub-cultured in William’s E Medium supplemented with 2 mM L-glutamine and 5% FBS in 96-well collagen I-coated microtiter plates and allowed to adhere overnight. The plating medium was then removed and replaced with a collagen overlay solution composed of 1500 µg/mL rat tail collagen I in NF, EMS, or LMS media, supplemented with HBSS 10X (at one-tenth the volume of the collagen solution) and 6 µL/mL of 1 M NaOH. The solution was mixed rapidly, and 50 µL of the gelling mixture was added to each well. The plates were incubated at 37 °C for 1 h to allow the collagen to polymerize, followed by the addition of 150 µL of media (NF, EMS, or LMS) per well. The cultures were maintained for an additional 6 days, with a single media exchange performed on Day 3. To assess bile canalicular function, a 2 mM stock solution of CellTracker™ Green CMFDA (InVitrogen) in DMSO was diluted 1:10 in media, then further diluted 1:100 directly into the hepatocyte culture media. The plates were incubated for 20–30 min, after which the CMFDA-containing medium was removed and replaced with fresh, untreated medium. Fluorescence was evaluated using an excitation/emission setting of 405/488 nm to assess bile canalicular flow.

### LipidTOX labeling and α-SMA Immunofluorescence

Lipid and α-smooth muscle actin (α-SMA) staining were performed following our previously established protocol [28]. Briefly, cells within the MPS devices were fixed with 4% paraformaldehyde (Thermo Fisher Scientific, AA433689M) in PBS for 30 min at room temperature. Fixation was followed by two 10-minute washes with PBS. For lipid staining and α-SMA labeling, LipidTOX™ Deep Red Neutral Lipid Stain (1:500; Invitrogen, H34477) and a mouse monoclonal anti-α-SMA antibody (1:100; Sigma-Aldrich, A2547) were perfused into the devices and incubated overnight at 4 °C. The following day, samples were incubated with a goat anti-mouse Alexa Fluor 488 secondary antibody (1:250; Invitrogen, A-11029) for 2 h at room temperature prior to imaging.

### Confocal imaging and analysis

Confocal imaging was performed using the Phenix High-Content Imaging platform (Revvity), using a 20×/0.4 hN A air objective. A z-stacks of 35-µm distance (5 μm spacing between slices) were obtained across an array of 7 × 15 adjacent fields covering an area of 44 mm^2^ in the LAMPS. Image analysis was performed as previously described [7].

### Quantification of steatosis

Steatosis was assessed at the conclusion of the experimental timeline on Day 8. Nuclei were stained with Hoechst 33,342 (1:4000; Invitrogen, R37605) and imaged using a 405-nm laser with a DAPI filter. Lipid accumulation was visualized using LipidTOX™ Deep Red, with fluorescence acquired via a 640-nm laser and Cy5 filter. Imaging parameters were as previously described [7].

### Quantification of stellate cell activation

Immunofluorescence for α-SMA expression in LX-2 cells was performed on Day 8. Nuclei were imaged using a 405-nm laser with a DAPI filter, and α-SMA was detected using a 488-nm laser with a FITC filter. Image analysis was conducted in Harmony software (Revvity, v5.1) using maximum intensity projections. α-SMA signal was thresholded and quantified as a percentage of total field area [7, 9].

### Efflux collection and functional analysis

Efflux samples from LAMPS were collected on days 2, 4, 6, and 8 to measure albumin (ALB), urea (BUN), lactate dehydrogenase (LDH), and pro-collagen Iα1 (COL1A1) levels, as previously described [9, 10, 27, 28]. ALB was quantified via ELISA (1:100 dilution; Bethyl Laboratories) using in-house human albumin standards. COL1A1 was measured using a commercial ELISA kit (R&D Systems, 1:50 dilution). BUN and LDH levels were assessed using Stanbio and Promega kits, respectively, adapted for 384-well plate format without dilution. For all assays media of the appropriate condition type and not exposed to cells was used as a blink control for background subtraction.

### Statistical analysis

One-way ANOVA was used to compare three or more studies conducted under identical conditions with the same cell lot, while a pooled t-test was applied for duplicate identical studies. The intraclass correlation coefficient (ICC) was used to assess both the intra- and inter-study reproducibility of the metrics measured at multiple time points evaluating both the magnitude and trend of the signals over time. Reproducibility of model performance is classified as excellent (ICC > = 0.8), acceptable (0.2 = < ICC < 0.8), or poor (ICC < 0.2). Inter-study reproducibility analysis was conducted using Eve Analytics™ following the Pittsburgh Reproducibility Protocol (PReP) [29, 30].

## Results

### Rigorous qualification of primary human cells for liver MPS integration

The FDA’s recent push to reduce reliance on animal testing has accelerated the development and adoption of microphysiological systems (MPS), including liver chip technologies, for both basic and translational research. These platforms offer promise in evaluating drug efficacy, safety, and patient stratification for clinical trials and therapeutic strategies. However, their successful integration into drug development workflows hinges on rigorous validation and reproducibility of performance particularly of the biological components. The reliability of any MPS platform depends critically on the source, quality, and functionality of the constituent primary cells.

Each of the cell types included in the liver MPS must retain defining characteristics of their in vivo counterparts. For hepatocytes, this includes being sourced from adult (≥ 18 years old) donors with demonstrated post-thaw viability and replating efficiency, commonly > 80% viability and > 70% replating. To model MASLD progression, hepatocytes must originate from phenotypically healthy donors, allowing for the controlled induction of disease under experimental conditions. Similarly, Kupffer cells or macrophage-like cells must express CD68 and respond to LPS with secretion of IL-1β, TNF-α, and IL-6. Hepatic stellate cells are validated by their responsiveness to TGF-β, as evidenced by increased proliferation, collagen Iα1 secretion, α-SMA expression, and morphological shift from oval shapes to cells with elongated projections [31]. LSECs must maintain permeability and fenestration, lacking tight junctions and basement membrane formation, to ensure physiological transport functions. In addition, we exclude donors with overt liver pathology and consider the presence or absence of genetic variants linked to liver disease susceptibility.

### Objectives and primary human hepatocyte selection criteria

The overarching goals of this study were threefold: (i) to develop a stepwise pipeline for evaluating the suitability of primary human hepatocyte (PHH) lots within the LAMPS platform; (ii) to compare performance across hepatocyte lots under physiologically relevant conditions; and (iii) to identify the most informative functional metrics aligned with MASLD modeling and drug testing. A core part of this process included establishing criteria for lot inclusion or exclusion based on age, health status, and genotype. Pediatric donors (< 18 years) were excluded due to differences in hepatic enzyme expression, metabolism, and regenerative capacity. Donors with known liver disease were also excluded to preserve the model’s capability to induce MASLD phenotypes. Additionally, lots were selected based on the presence or absence of genetic variants relevant to the study’s goals.

To meet these objectives, we implemented a structured, phased assessment framework (Fig. 1), which we applied to four hepatocyte lots. This approach enabled a robust evaluation of reproducibility, performance, and suitability for MASLD-focused applications in LAMPS.

### Phase I: initial cell viability and morphology assessment

**Fig. 2:**
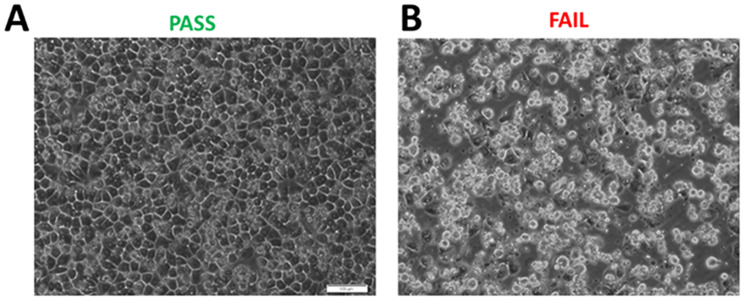
Brightfield imaging of hepatic cell seeding in MPS Pass / Fail. **(A)** 4–24 h after seeding of the MPS with hepatic cells they should show a cobblestone morphology with pronounced bright borders where cell-to-cell contacts occur to receive a passing score for cell attachment. **(B)** Example of a hepatic lot of primary human cells that failed to show strong adhesion even after a 24-hour attachment period

**Fig. 3:**
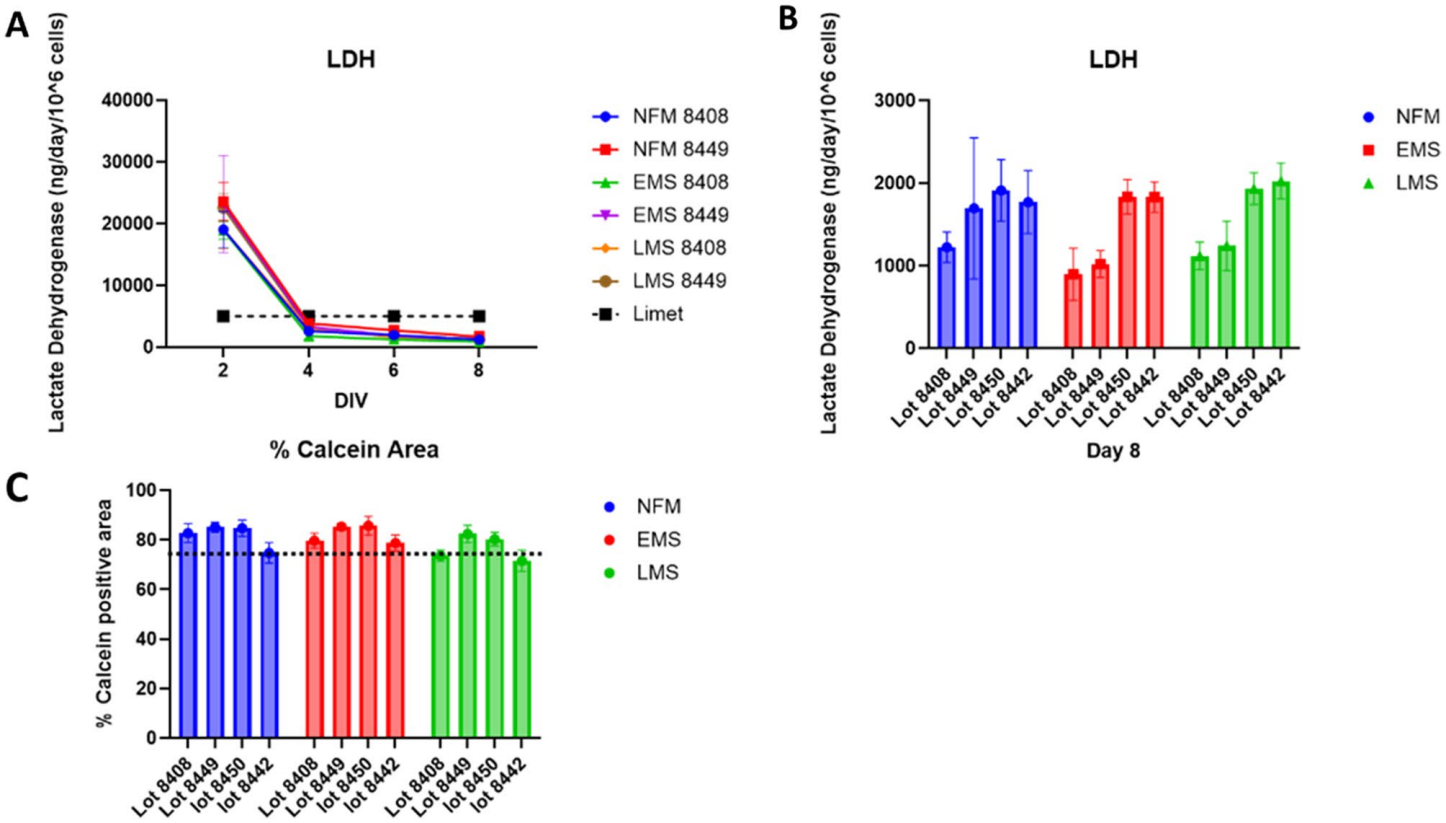
Evaluating cell survival in MPS device. **(A)** Example of two lots of hepatocytes cultured in three different media types show a drop in LDH over time and remaining below the limit of 5000 nanograms per day per million cells after day two *N* = 7. **(B)** Comparison between different primary hepatic lots and their LDH levels at the conclusion of microphysiological organ culture on day 8 as an effect of media type. No significant effect of media type was found though primary lot could have a significant impact on LDH secretion *N* = 7 *p* ≥ .05. **(C)** Live cell Calcein imaging quantification after eight days in culture of liver MPS to evaluate cell survival and MPS coverage at the close of the experiment *N* = 7 for each condition Calcein coverage of 75% or greater is the cut off for NFM with small effects being permissible for disease medias such as EMS and LMS

Initial testing began with basic trypan blue viability assays to assess cell health, alongside evaluations of cell attachment and morphology. As shown in (Fig. 2), hepatocyte lot 1045 demonstrated excellent attachment 24 h post-seeding, exhibiting classic cobblestone morphology and clearly defined cellular borders indicative of strong cell-cell connections. In contrast, lot 1133 showed poor attachment and abnormal morphology at the same time point. Although we do not adhere to strict replating criteria, we typically proceed with further testing for lots that show > 80% viability post-thaw and exhibit cell attachment and spreading within 8 h. Lots meeting these thresholds are considered replateable and advance to the next phase, where the full LAMPS biomimetic model is established. This involves seeding three non-parenchymal cell types and initiating media flow, which is maintained for 8 days. Effluent media is collected every 48 h to assess secretome output, providing key insights into the overall health and functionality of the LAMPS (Fig. 3).

### Phase II: viability and stability metrics

Lot assessment includes two primary cell viability metrics (Fig. 3). LDH release is monitored over the full 8-day culture period to assess temporal cell health, while Calcein-AM staining at endpoint provides a direct measure of live, metabolically active cells. In trials using two hepatocyte lots across three media types, we observed an initial spike in LDH release as the model stabilized, typically by Day 4, followed by a consistent low-level release (< 2500 ng/day/10⁶ hepatocytes) for the remainder of the experiment (Fig. 3A). This threshold was defined based on historical data [Supplemental Table 1]. At endpoint, chips were perfused with Calcein-AM, and cell viability was quantified by measuring the area of positive staining across all four liver cell types in the model (Fig. 3B). We consider ≥ 80% ± 3% Calcein-AM positivity as acceptable across all media types, as below this point, we saw a reduction in albumin production as well as reduced cell to cell border interactions indicating that the cells were no longer operating together as a micro-organ. For comparison, Fig. 3C shows LDH levels on the same day that Calcein-AM staining was assessed. While all four lots met the LDH release criteria, lot 8442 was ultimately rejected due to failing the Calcein-AM metric under LMS conditions, underscoring the importance of multi-parametric acceptance criteria.

### Phase III: performance of hepatic functional assays

**Fig. 4:**
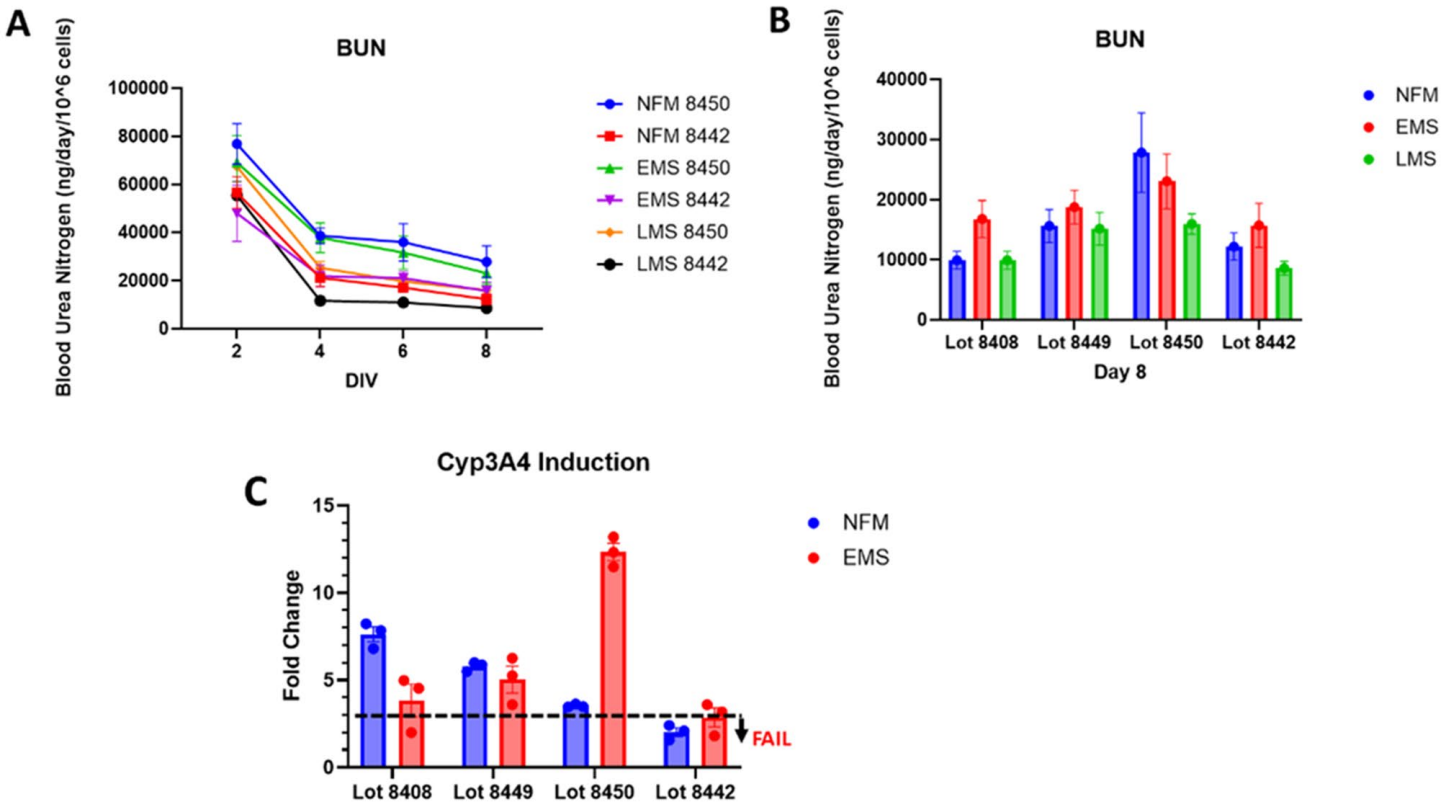
Liver urea production and CYP induction. **A**) Urea production in liver MPS over the 8-day time course *N* = 7. **(B)** Lot to lot variation in urea production at the conclusion of 8-day experiment *N* = 7 No significant effect via media was observed though there was a significant effect of hepatic lot. **(C)** CYP3A4 induction by rifampicin should show a greater than or equal to threefold increase after drug exposure

Our LAMPS functional assays cover several aspects of liver function, including protein synthesis (albumin), ammonia detoxification and mitochondrial activity (via urea production), hepatocellular transport (bile canalicular formation and efflux), and metabolic enzyme induction (Cyp3A4 activity following rifampicin treatment). Urea production for two hepatocyte lots across three media conditions is shown in (Fig. 4A). Comparing all 4 test lots on day 8 of media condition exposure (Fig. 4B). The urea cycle is notably influenced by media composition, especially under LMS-induced disease progression conditions. Compared to historic urea values [Supplemental Table 1] our acceptance threshold for urea production is ≥ 20,000 ng/day/10⁶ hepatocytes between days 2–8 in NF and EMS conditions as this value more consistently represent primary hepatocyte output where the morphology and overall health of the cells appears functional without increased disease markers such as excessive lipid accumulation [9, 26]. Among tested lots, lot 8450 demonstrated the strongest urea output based on these criteria. Note that the appropriate media not exposed to the cells was used as a blank control such that the values reported reflect the effects of cellular process though it is worth noting that this is relative measure of hepatocyte metabolic activity rather than a direct readout of urea cycle flux.

To assess oxidative metabolism, we conducted plate-based CYP3A4 activity assays following 48-hour treatment with 10 µM rifampicin as this is a critical CYP for a great deal of the drug metabolism (Fig. 4C). A minimum three-fold induction post-treatment is required for acceptance. All but one lots met this standard. Notably, two lots showed higher CYP3A4 induction under NF conditions, while the remaining two responded more robustly under EMS, reflecting lot-specific variability.

**Fig. 5:**
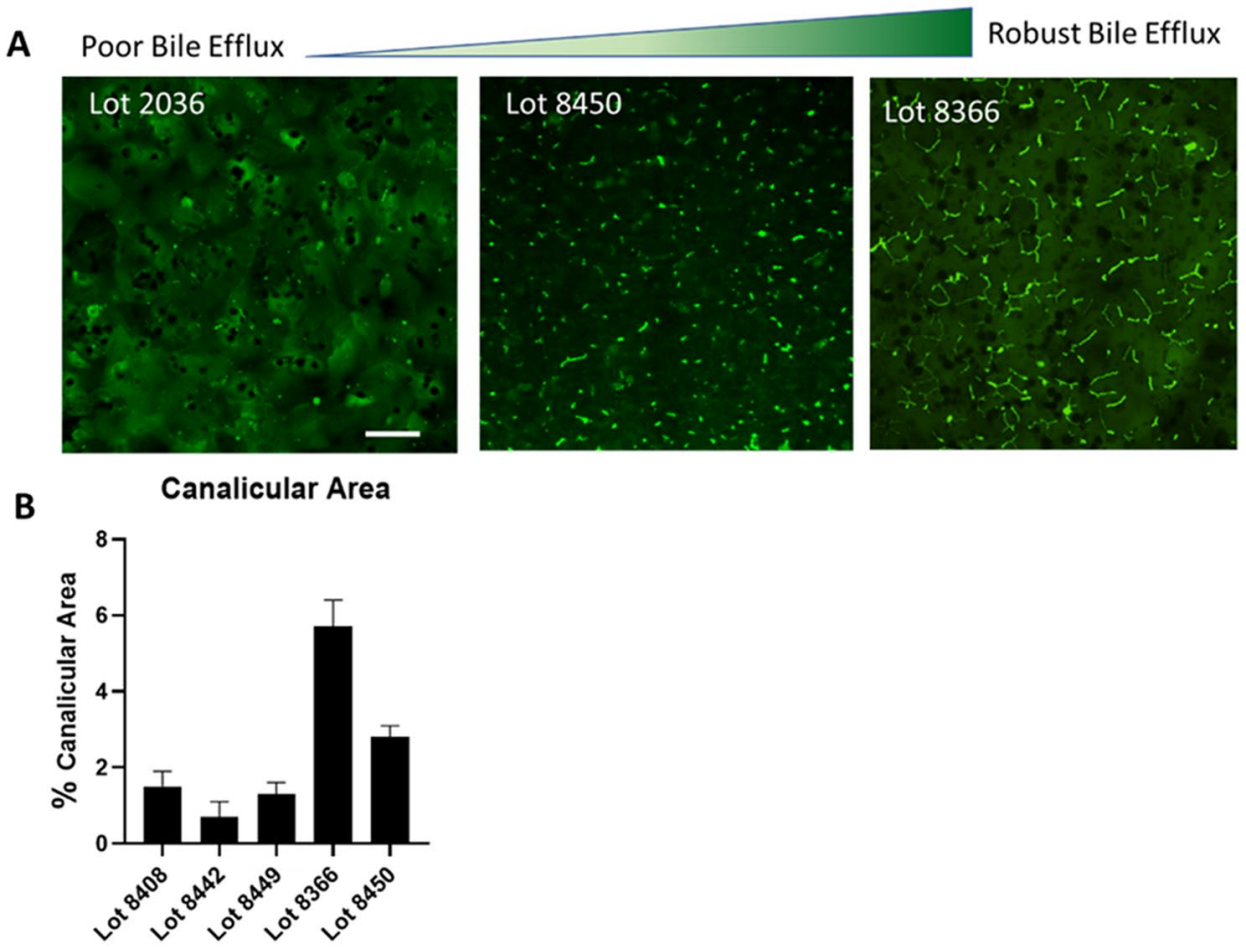
Bile efflux **(A)** Calibrating images for bile efflux evaluation from poor (Canalicular area <%2) to robust (Canalicular area >%5). scale bar 50 μm. **(B)** Quantification of canicular area I hepatocyte lot DIV3

Bile canalicular activity, a marker of hepatocellular polarization and transport processes, was assessed on day 5 or 6 using CMFDA staining under a collagen overlay (Fig. 5A). Response varied from weak to strong among lots. We accept quantification ≥ 2% image area with canalicular activity constitutes an acceptable performance (Fig. 5B).

**Fig. 6:**
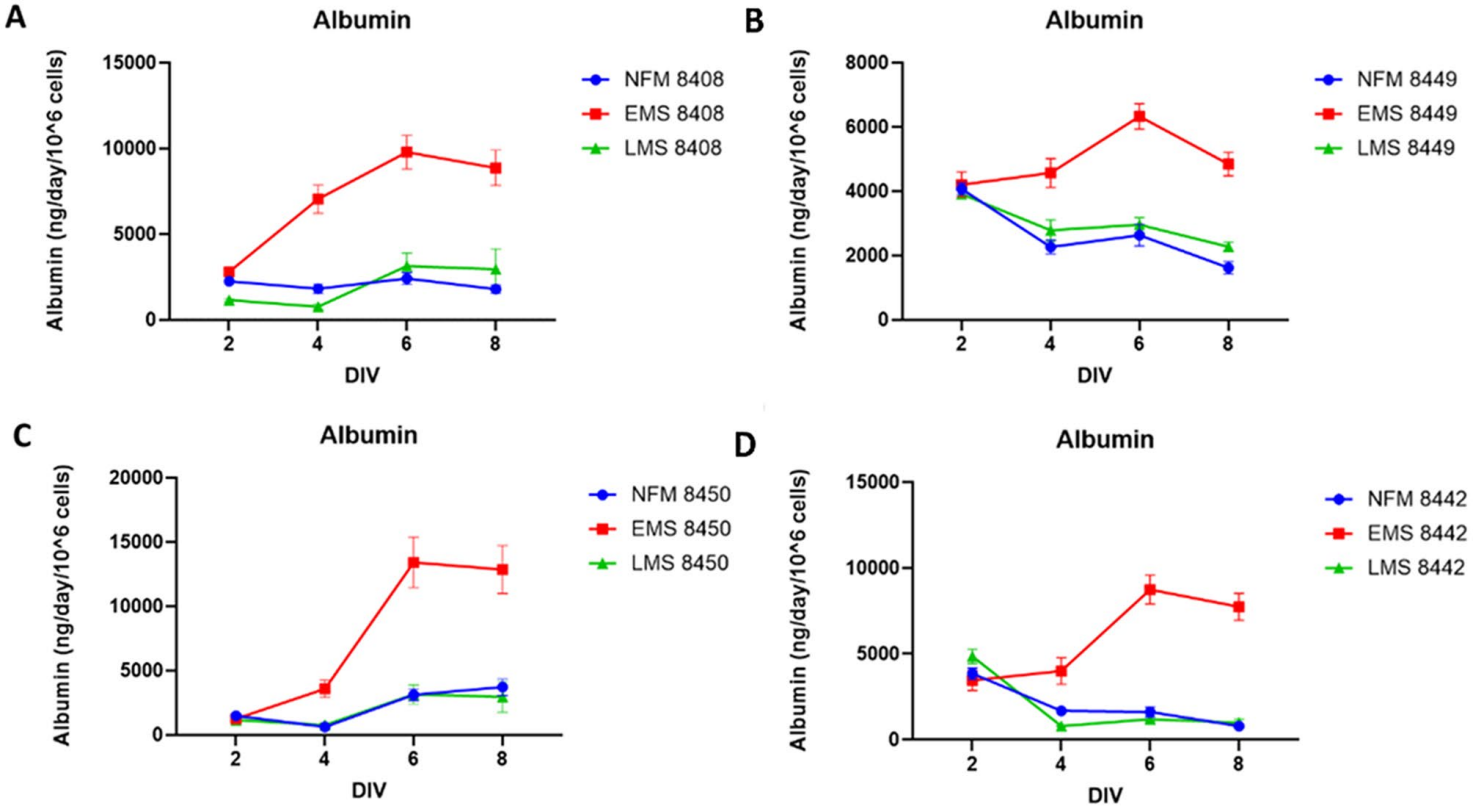
Effects of disease progression on albumin production. **A-D)** Albumin production in liver MPS over 8-day time course with normal fasting early stage and late-stage metabolic syndrome formulated media. EMS is significantly elevated over that of NF or LMS p = ≤ 0.05, *N* = 7) For four different lots of primary Hepatocytes

Albumin production represents our fourth functional assay (Fig. 6), and based on historical data, it is arguably the most sensitive indicator of hepatotoxicity and MASLD progression [4, 9, 10, 26–28]. Across four tested lots, EMS media consistently supported the highest albumin output compared to NF and LMS (Fig. 6A–D). Compared to historic albumin values [Supplemental Table 1], the lots exhibited the expected profile, with peak production between days 5–7 followed by gradual decline. While overall albumin levels across lots and media types remain relatively consistent, the specific time-course and magnitude of peak production are lot-dependent.

### Phase IV: MASLD phenotype induction

**Fig. 7:**
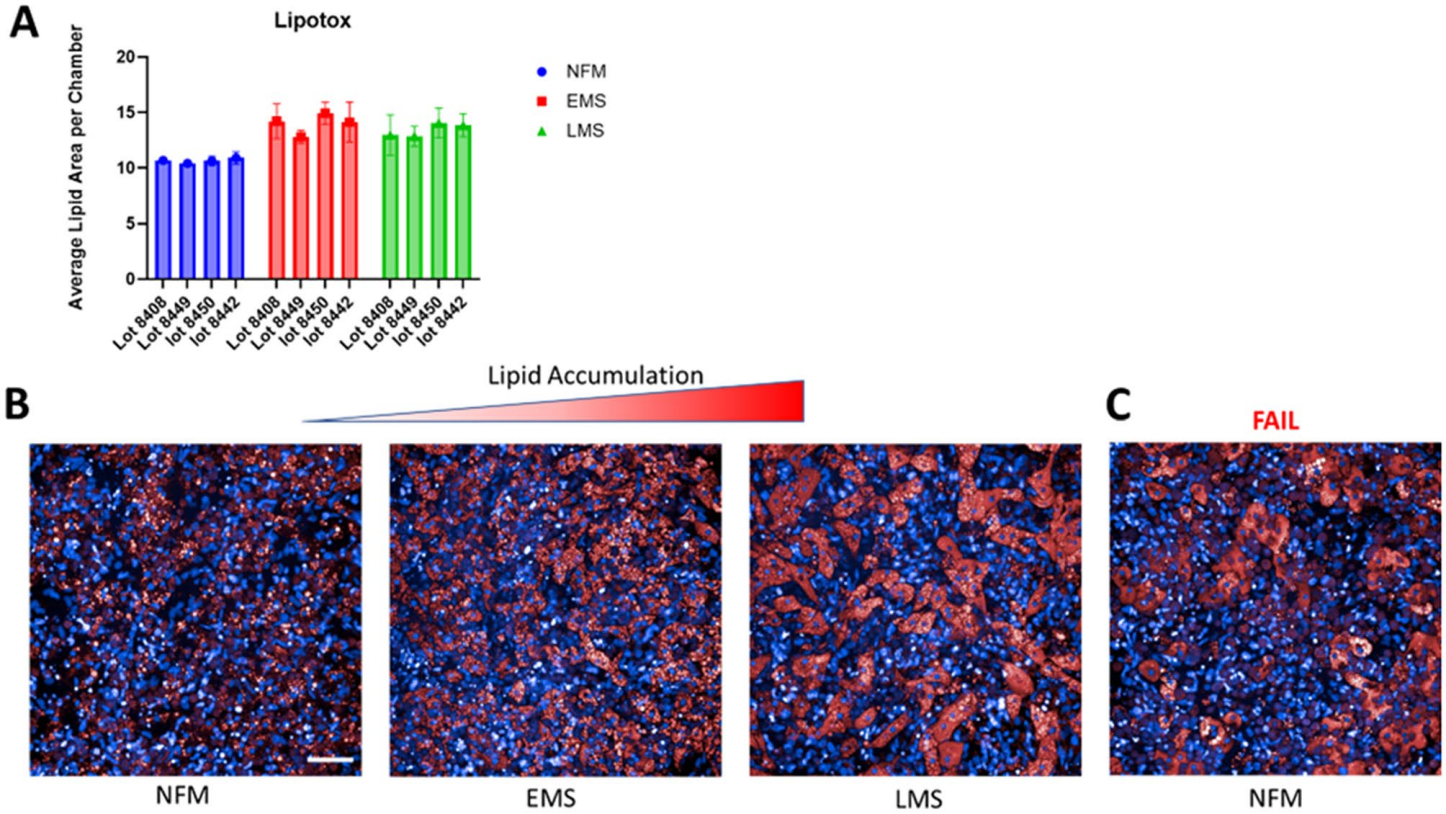
Induction of disease induced Steatosis in liver MPS model influenced by hepatic lot. **(A)** Lipid accumulation quantification in liver MPS over 8-day time course with normal fasting, early stage and late-stage metabolic syndrome formulated media. EMS and LMS is significantly elevated over that of NF p = ≤ 0.05, *N* = 7) For four different lots of primary Hepatocytes. **(B)** Representative images of the expected lipid accumulation as a result of disease progression. An average lipid area per chamber below 11 was considered indicative of poor lipid accumulation, while values above 14 were considered high. **(C)** Abnormally high lipid accumulation in normal fasting media is a potential metric for excluding hepatic cell lot selection for normal healthy tissue. scale bar 50 μm

To model MASLD progression, we assessed steatosis and fibrosis-two hallmarks of disease progression- in all lots under NF, EMS, and LMS conditions. These include pro-fibrotic drivers like TGF-β1 and LPS as was previously established [9]. Steatosis was quantified by lipid accumulation using lipidTOX staining (Fig. 7). Based on Fig. 7, EMS and LMS treatments resulted in significant increases in lipid content, meeting our acceptance criteria. However, some lots, such as 8408, failed to exhibit significant lipid accumulation in LMS, despite doing so in EMS (Fig. 7A). In contrast, the remaining three lots met the lipid-based acceptance thresholds under both conditions.

**Fig. 8:**
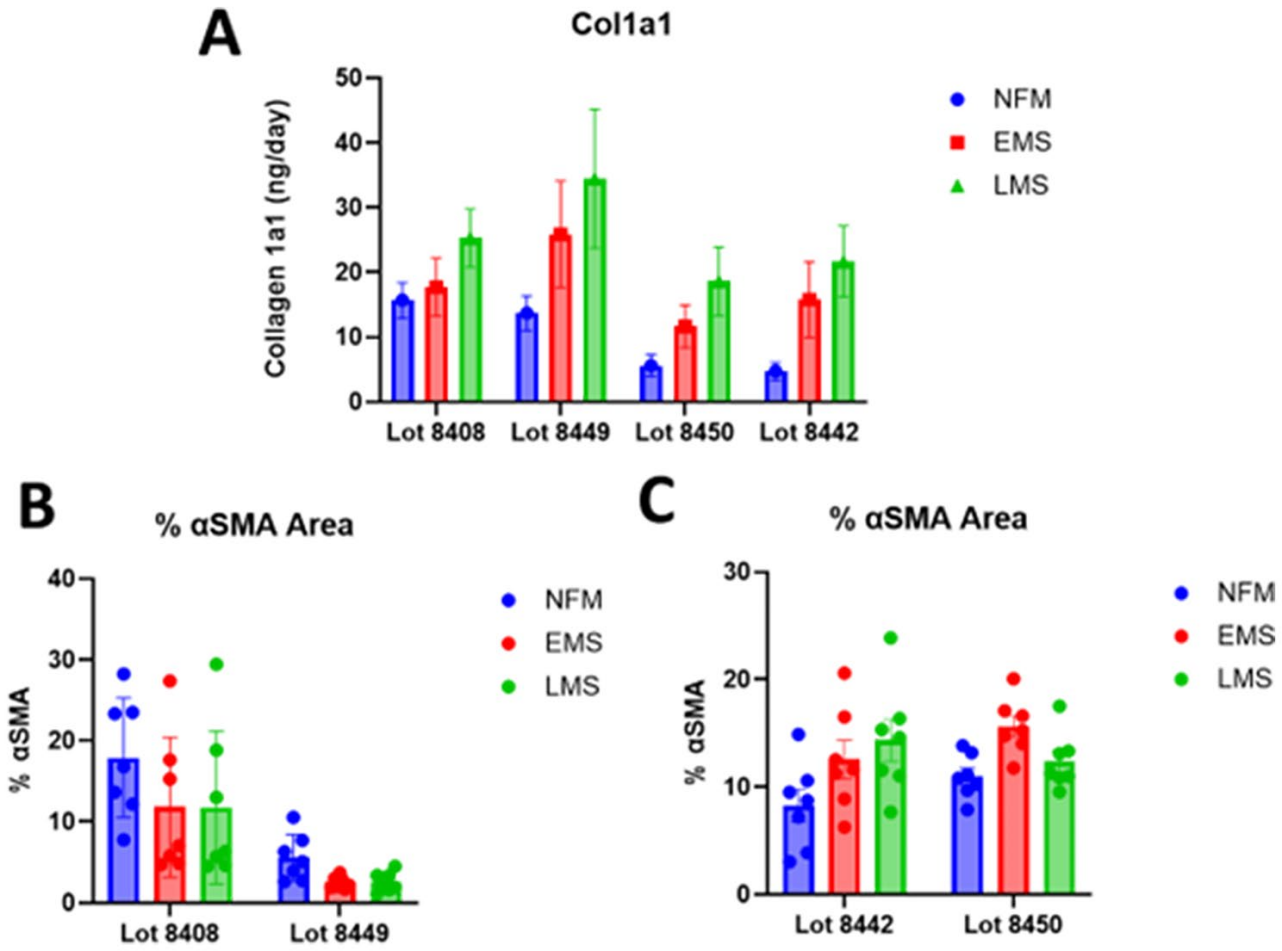
Induction of disease induced fibrotic changes in liver MPS model influenced by hepatic lot. **A**) COL1A1 production is associated with stellate cell activation as a result of early and late-stage metabolic syndrome in three of the four lots tested showed significant COL1A1 production increase as a result of media except lot 8408. **B)** Neither lot of hepatocytes showed an increase in percent α-SMA staining as a result of metabolic syndrome induction. **C)** Both lots showed a trend toward increased α-SMA as a result of metabolic syndrome induction with lot 8450 being statistically significant

Fibrosis, the second MASLD hallmark, is assessed by measuring pro-collagen Iα1 (COL1A1) secretion into the media (Fig. 8A). Lots 8443 and 8450 exhibited the clearest distinction in COL1A1 levels over time. LAMPS are also fixed on Day 8 for immunofluorescence staining of α-SMA to assess stellate cell activation (Fig. 8B, C). However, α-SMA has proven to be a less reliable fibrosis marker, heavily influenced by hepatocyte lot. For instance, lots 8408 and 8449 did not show increased α-SMA staining, whereas 8442 and 8450 did, though only lot 8450 in EMS reached statistical significance.

### Phase V: reproducibility testing

**Fig. 9:**
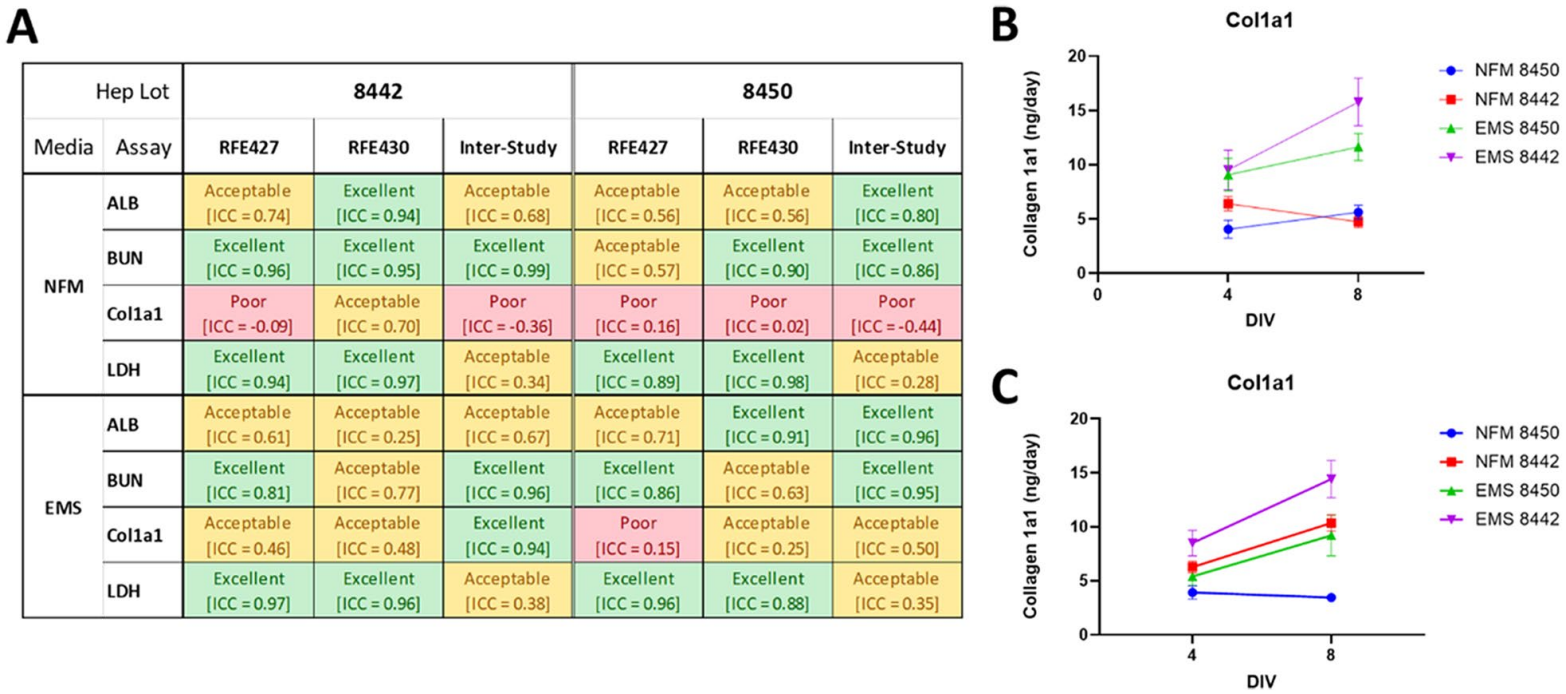
Overall, acceptable reproducibility was observed with albumin, urea, LDH and COL1A1 secretion using the two lots that showed the greatest promise for MPS platform. **(A)** Analysis of intra and inter-study reproducibility for the two remaining lots that showed the greatest promise for inclusion in our MPS platforms. **(B)** First run of liver MPS looking at COL1A1 production *N* = 7 showed good separation for both lots between NF and EMS conditions. **(C)** Second run of liver MPS looking at COL1A1 production *N* = 7 lot 8442 showed reduced separation between NF and EMS conditions trend remained

Final lot selection is based not only on performance metrics but also on reproducibility. The top two candidates from initial testing are re-evaluated to ensure consistent results. Both lots demonstrated acceptable reproducibility (Fig. 9A), with lot 8450 showing slightly superior consistency, except in COL1A1 levels under NF. However, this discrepancy is not particularly concerning, as collagen production under non-inflammatory conditions (e.g., NF) remains low and stable from Day 4 to 8, as expected. Graphs of COL1A1 from both two independent runs (Fig. 9B, C) confirm this stability in lot 8450, reinforcing the reproducibility of the data.

## Discussion

The rapid growth of the field of microphysiological systems (MPS) has accelerated in part by recent FDA directives to shift away from animal studies in favor of human-based models, enabled the development of more physiologically relevant in vitro systems for studying complex organ functions and evaluating therapeutic interventions. As these platforms gain traction across academic and pharmaceutical research, the quality and suitability of the cells used to populate them have become central to their success. With a growing diversity of cell sources including primary human hepatocytes (PHHs), induced pluripotent stem cell-derived hepatocytes (iPSC-Heps), and hepatic cell lines such as HepG2 there is an urgent need for structured, reproducible strategies to assess cell fitness tailored to specific research objectives [32].

While physiologically relevant benchmarks provide valuable tools for assessing MPS functionality, relying solely on rigid performance metrics may overlook intrinsic biological variation due to donor genetics, disease state, or patient-specific factors. Such limitations can obscure meaningful biological insights and hinder reproducibility across studies. To address these challenges, a more nuanced and adaptable qualification process is needed one that balances functional relevance with biological heterogeneity and enables rigorous, yet flexible, evaluation of cell sources. This approach ensures that selected cells not only meet baseline performance standards but also capture the complexity of human biology, thereby enhancing the translational value of MPS platforms. To address this, we have developed and implemented a systematic structure that defines a phased, multi-parameter evaluation process for qualifying hepatic cell sources for use in liver MPS, particularly those used to study MASLD. This structure evaluates cell parameters such as plating efficiency, viability, liver-specific functionality (e.g., urea, albumin, and CYP450 activity), and the ability to demonstrate key disease phenotypes (e.g., steatosis, inflammation, fibrosis).

When it comes to MASLD modeling, it’s important to recognize that no model can fully capture all the complexities of the human disease. However, this does not diminish the model’s value for research or clinical applications; it simply highlights the need to acknowledge its limitations. In this study, since we’re focusing on selection criteria rather than a comprehensive characterization, our goal is to identify the minimum set of tests needed to appropriately select hepatocyte lots for more extensive MPS experiments. For example, a hepatocyte lot that already shows significant lipid accumulation in normal fasting media (likely due to the donor’s lifestyle) would be a poor candidate for MASLD modeling, as it’s unlikely that additional lipids would further drive the phenotype the steatosis may already be at its maximum. This doesn’t mean that the same hepatocyte lot couldn’t be valuable for other MPS testing, which is why we propose a “fit for purpose” evaluation framework in this manuscript.

While this cell selection pipeline is primarily focused on hepatocytes, it’s important to acknowledge the role that stellate cells play in the model. A key component of this MPS is LX-2, an immortalized stellate cell line, which offers the advantage over primary stellate cells of remaining quiescent even after being transferred into the device, as indicated by minimal α-SMA expression [33, 34]. Stellate cells are essential drivers of fibrosis [35–37] and are the main source of collagen 1A1 production [35, 36]. Therefore, while selecting the stellate cells for your MPS is critical for assessing fibrosis, this aspect was beyond the scope of our manuscript. Our focus here was on how the hepatocyte lot interacts with the chosen LX-2 cells to either activate them or not, influencing the development of MASLD-related fibrosis.

A key feature of this structure is its emphasis on establishing a “fit-for-purpose” selection strategy matching the model’s design and goals with the specific attributes of the cells. This approach promotes reproducibility and robustness within and across labs, while minimizing the risk of experimental variability that could obscure biologically meaningful findings. Importantly, however, fit-for-purpose optimization can become a double-edged sword if it inadvertently biases against capturing intrinsic population heterogeneity. Biological variability whether due to genetic background, disease susceptibility, or donor history is highly relevant to both disease modeling and drug response studies.

Therefore, our structure explicitly incorporates the evaluation of multiple hepatocyte lots, enabling the systematic identification of how intrinsic donor heterogeneity impacts MPS outcomes. For instance, assessing how different lots respond to metabolic stress or fibrotic stimuli helps reveal variability in disease progression trajectories critical meta information for advancing precision medicine approaches. This level of characterization not only supports internal rigor but also facilitates broader standardization and data comparability across institutions and research consortia.

Given that primary hepatocyte lots are finite and non-renewable, our structure further serves as a benchmark framework for evaluating and comparing patient-derived iPSC hepatocytes. By generating rigorous, quantitative performance metrics from PHHs, we create a valuable reference for assessing the maturation and disease modeling capabilities of iPSC-Heps, enhancing their translational potential.

Moreover, embedding quality control and functional assessment into the cell selection process such as albumin production, CYP induction, and fibrosis induction ensures that liver MPS platforms are both physiologically relevant and capable of modeling disease states such as MASLD. These standardized practices strengthen the foundation for collaborative science and increase confidence in the biological conclusions drawn from MPS-based studies.

Future directions should look at optimizing MPS systems for compatibility with multi-omic approaches as well as flow sorting so that cell type specific omic data could be retrieved from the MPS. As these systems are often within closed devices and often contained collagen matrix components as well as a smaller number of cells than is typical of well plate assays; optimizing protocols for extraction efficiency and for detection sensitivity will be needed for combining these modalities effectively moving forward.

In conclusion, the implementation of a structured, reproducible roadmap for cell qualification in liver MPS is critical for advancing the field toward more predictive, translatable, and clinically meaningful models. As MPS technologies continue to evolve, fostering cross-platform consistency while embracing biologically relevant variability will be essential for unlocking new mechanistic insights and improving therapeutic development.

## Supplementary Information

Below is the link to the electronic supplementary material.

Supplementary Material 1

Supplementary Material 2

Supplementary Material 3

## Data Availability

The datasets used and/or analysed during the current study are available from the corresponding author on reasonable request.
