# Supplementary figures and images for: Road map for primary hepatocyte qualification in human liver organ models
Source: BMC Methods. 2026 Feb 9;3(1):6. doi: 10.1186/s44330-026-00058-7 (PMC12883510; doi:10.1186/s44330-026-00058-7)

Sup Figure 1.

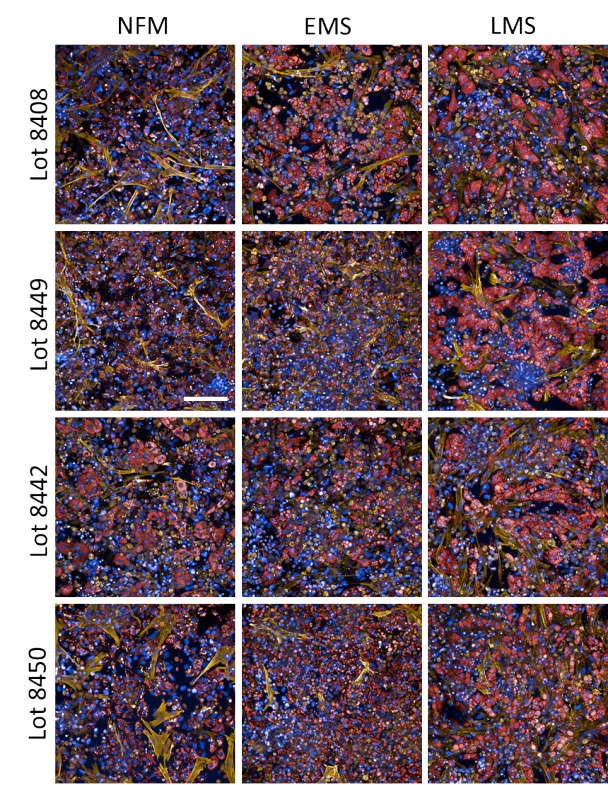

Supplement: Supplementary file 1 — Supplementary Material 1 [file 44330_2026_58_MOESM1_ESM.pdf]

Supplemental figure 2

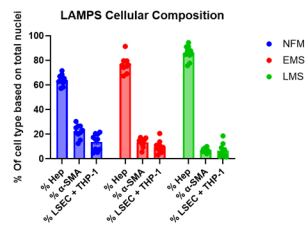

Supplement: Supplementary file 3 — Supplementary Material 3 [file 44330_2026_58_MOESM3_ESM.pdf]
