# Supplementary material for: Road map for primary hepatocyte qualification in human liver organ models
Source: BMC Methods. 2026 Feb 9;3(1):6. doi: 10.1186/s44330-026-00058-7 (PMC12883510; doi:10.1186/s44330-026-00058-7)
Supplement: Supplementary file 2 — Supplementary Material 2 [file 44330_2026_58_MOESM2_ESM.pdf]

Sup Table 1.

Lot	LDH output daily	LDH output peak	Albumin output daily	Albumin output peak	Urea output daily
8391	1071 ± 500	3662 ± 1596	9708 ± 2098	13157 ± 1878	31113 ±5234
8450	2372 ± 245	11414 ± 3506	5520 ± 1529	11414 ± 3110	40467 ±5460
912	1138 ± 277	6262 ± 1798	1495 ± 348	2059 ± 459	23446 ±3204
1181	1214 ± 166	8991 ± 893	3292 ± 458	4087 ± 504	34925 ±939
8442	2446 ± 419	4866 ± 331	2315 ± 351	2938 ± 890	35417 ±7330
1183	1004 ± 262	5050 ± 416	10416 ± 3914	13920 ± 9094	22493 ±2685
658	638 ± 257	2094 ± 422	3263 ± 541	4563± 844	19826 ±230
1142	186 ± 70	401 ± 104	13863 ± 3243	20441 ± 1455	36063 ±1763
